# Ac_2–26_ Hydrogel Modulates IL-1β-Driven Inflammation via Mast Cell-Associated and Immune Regulatory Pathways in Diabetic Wounds

**DOI:** 10.3390/cells14130999

**Published:** 2025-06-30

**Authors:** Monielle Sant’Ana, Rafael André da Silva, Luiz Philipe S. Ferreira, Cristiane D. Gil, Fernando L. Primo, Ana Paula Girol, Karin V. Greco, Sonia M. Oliani

**Affiliations:** 1 Structural and Functional Biology Graduated Program, Federal University of São Paulo (UNIFESP), São Paulo 09913-030, SP, Brazil; monibiologia@yahoo.com.br (M.S.); luiz.philipe@unifesp.br (L.P.S.F.); cristiane.gil@unifesp.br (C.D.G.); anapaula.girol@unifipa.com.br (A.P.G.); k.greco@ucl.ac.uk (K.V.G.); 2 Department of Biology, School of Biosciences, Humanities and Exact Sciences, São Paulo State University (UNESP), São José do Rio Preto 15054-000, SP, Brazil; rafaelandre.dasilva@cuanschutz.edu; 3 Department of Engineering of Bioprocess and Biotechnology, School of Pharmaceutical Sciences, São Paulo State University (UNESP), Araraquara 14800-903, SP, Brazil; fernando.primo@unesp.br; 4 Experimental and Clinical Research Center (CEPEC), Padre Albino University Center (UNIFIPA), Catanduva 15809-144, SP, Brazil; 5 Department of Surgical Biotechnology, Division of Surgery & Interventional Science, Royal Free Hospital, School of Medicine University College London (UCL), London NW3 2PF, UK; 6 Advanced Research Center in Medicine (CEPAM), União das Faculdades dos Grandes Lagos (Unilago), São José do Rio Preto 15030-070, SP, Brazil

**Keywords:** diabetes, wound healing, mast cell, IL-1β, annexin A1

## Abstract

Chronic, non-resolving inflammation is a major contributor to impaired wound healing in diabetes. Annexin A1 (AnxA1), a pro-resolving mediator, and its mimetic peptide Ac_2–26_ have demonstrated therapeutic potential in modulating inflammatory responses. In this study, we evaluated the effects of topical Ac_2–26_ hydrogel in a streptozotocin-induced diabetic wound model. Treatment significantly accelerated wound closure, improved tissue architecture, and reduced leukocyte infiltration. Immunohistochemical analysis revealed diminished mast cell accumulation and IL-1β expression in treated wounds. Complementary transcriptomic profiling supported the downregulation of pro-inflammatory genes, including Il1b and mast cell-related mediators, confirming the peptide’s regulatory effect on the wound immune landscape. Mounting evidence suggests that dysregulated mast cell activity plays a role in the heightened inflammatory tone and delayed tissue repair observed in diabetic wounds. In our model, Ac_2–26_ hydrogel treatment attenuated IL-1β expression, suggesting an indirect downregulation of NLRP3 inflammasome activation, potentially mediated through mast cell modulation, though effects on other cell types within the wound microenvironment cannot be excluded. While definitive causality cannot be assigned, the integration of histological and transcriptomic data highlights mast cells as contributors to the IL-1β-driven inflammatory burden in diabetic wounds. These findings underscore the immunomodulatory capacity of Ac_2–26_ and its potential to restore resolution pathways in chronic wound settings, positioning it as a promising candidate for future therapeutic development.

## 1. Introduction

Type 1 diabetes mellitus and its complications are characterized by immune system dysregulation and persistent low-grade inflammation. Among the various immune cells involved, mast cells (MCs) have gained recognition as key participants in both the onset and progression of diabetes-related pathologies [1,2,3]. In this context, MCs contribute to the inflammatory milieu within pancreatic islets, promoting β-cell destruction through the release of histamine, cytokines, and chemokines, thereby sustaining immune cell activation and recruitment [4]. This evidence supports the multifaceted role of MCs not only in the pathogenesis of type 1 diabetes mellitus but also in the chronic inflammatory complications that accompany it [5].

Dogma dictates that MCs are also deeply involved in tissue repair processes and are implicated in all stages of wound healing. In diabetes, however, their dysregulated activation contributes to the pathogenesis of chronic diabetic foot ulcers and delayed tissue regeneration [2,5,6]. Impaired wound healing remains a major cause of morbidity and hospitalization among diabetic patients. It is estimated that 10–15% of these individuals will develop foot ulcers, with approximately 15% requiring lower limb amputation, and both scenarios are associated with high one-year mortality rates of 16.7% [7,8]. The hyperglycemic microenvironment promotes the accumulation of advanced glycation end products (AGEs), which in turn contribute to aberrant mast cell activation and the amplification of chronic inflammation in wound sites [2].

Once activated, MCs release a variety of pro-inflammatory mediators, including interleukin-1β (IL-1β), a key cytokine involved in the perpetuation of inflammation and impaired wound resolution. IL-1β production may occur through inflammasome-dependent or -independent pathways. One well-described mechanism involves the activation of the NOD-like receptor family pyrin domain containing 3 (NLRP3), a cytosolic sensor capable of forming the inflammasome complex that mediates caspase-1 activation and IL-1β maturation [9,10,11]. Although dysregulation of the NLRP3 inflammasome has been implicated in various chronic inflammatory and metabolic diseases [10,12,13,14,15,16], its precise role in diabetic wound healing remains unclear and probably intersects with other inflammatory cascades.

Annexin A1 (AnxA1), a protein endogenously induced by glucocorticoids, has emerged as a relevant modulator of inflammation and immune cell activation. AnxA1 and its N-terminal mimetic peptide Ac_2–26_ (AnxA1_2–26_) exert potent anti-inflammatory effects by suppressing eicosanoid synthesis and phospholipase A2 (PLA2) activity, thereby modulating the inflammatory response at different levels [17]. The role of AnxA1 has been extensively explored in our laboratory, demonstrating its protective action in both acute and chronic inflammatory models [18,19,20,21,22,23]. Previous work from our group using a second-degree burn model treated with 1% silver sulfadiazine (SDP 1%) showed modulation of MC profile and AnxA1 expression throughout the healing process [24]. More recently, we demonstrated that topical application of Ac_2–26_ hydrogel on diabetic wounds promoted reduced inflammatory infiltrates and improved healing outcomes [25].

Building on these findings, the present study aimed to investigate the effects of AnxA1_2–26_ hydrogel on mast cell modulation and IL-1β expression during the cutaneous wound healing process in diabetic mice. Additionally, NLRP3 expression was assessed as a supplementary marker of inflammatory signaling, providing further insights into the local immune response.

## 2. Materials and Methods

### 2.1. Ac_2–26_ Hydrogel Production

We produced an annexin A1_2–26_ hydrogel (Ac_2–26_) and evaluated the UV–vis absorption and fluorescence emission profiles of both the free peptide and the peptide incorporated with the hydrogel [25]. Briefly, the analytical quantification curve was determined using the UV–vis absorption method, UV–vis fluorescence emission, and a cytotoxicity assay that included the resazurin test. The Ac_2–26_ (2 µM) was incorporated into a carboxymethylcellulose (CMC) hydrogel. The vehicle hydrogel was prepared using CMC but without Ac_2–26_.

### 2.2. Animals

Male C57BL/6 mice (6–8 weeks old, 20–30 g) were obtained from UNIFIPA’s Research Unit and divided into 12 groups of 5 animals. They were housed in temperature-controlled cages (24–25 °C) with free access to food and water. All procedures followed ethical guidelines approved by CEUA-UNIFESP (Certificate 3958220419) and CEUA-UNIFIPA (Certificate 01/19).

### 2.3. Induction of Type 1 Diabetes Mellitus (DM1)

To induce DM1, animals underwent intraperitoneal (i.p.) injections of streptozotocin (STZ) at 65 mg/kg for five days following a 6 h fast. STZ was prepared in sodium citrate buffer (300 µL per dose). Control animals received only citrate buffer. Blood glucose levels were measured the day after the final injection using a glucose monitor, with samples taken from the tail. Animals with glucose levels ≥300 mg/dL were included in the experiment [26]. Non-responding animals were subject to repetition of the procedure or were euthanized if necessary.

### 2.4. Experimental Skin Injury Procedure and Pharmacological Treatment

To create the lesion, animals were anesthetized with intraperitoneal ketamine (100 mg/kg) and xylazine (10 mg/kg). The dorsal area was shaved, cleaned, and disinfected with Betadine^®^ and 70% alcohol. An 8 mm punch biopsy tool was used to generate the lesion, which was then immediately treated with either AnxA1_2–26_ peptide hydrogel or a vehicle hydrogel (without AnxA1_2–26_). The hydrogel was applied directly onto the dorsal wound surface of each animal using a sterile spatula. After application, the animals were carefully monitored for at least 1 h to ensure the hydrogel remained in place and to prevent behaviors that could compromise the integrity of the treatment.

The wound-healing effectiveness of AnxA1_2–26_ was tested in non-diabetic and diabetic mice. Non-diabetic mice were split into two subgroups: ND-V (treated with vehicle hydrogel) and ND-A (treated with AnxA1_2–26_ hydrogel), with 30 mice in each. Similarly, diabetic mice were divided into D-V (vehicle hydrogel) and D-A (AnxA1_2–26_ hydrogel) subgroups, also with 30 mice each. Treatments involved daily application of AnxA1_2–26_ hydrogel (2 µM) or vehicle hydrogel for 3 or 14 days. Wound healing was monitored, and animals were euthanized at the end of each period for lesion collection and analysis.

### 2.5. Histopathology Analysis

The wound site was observed daily to monitor inflammation, healing, and signs of infection, such as purulent areas or necrosis. Skin samples were fixed in 4% paraformaldehyde for 24 h, dehydrated in graded ethanol, cleared with xylene, and embedded in paraffin. Sections (3 µm) were cut using a Leica RM 2265 microtome (Wetzlar, Germany) and stained with hematoxylin-eosin. The analyses were conducted using a ZEISS AXIOSKOP2 microscope (Jena, Germany) and AXIOVISION software (Oberkkochen, Germany, version 4.8) at the Immunomorphology Laboratory, IBILCE-UNESP.

#### 2.5.1. Quantification of Mast Cells

For quantification of mast cells (MCs), immature or intact cells were characterized by metachromatic cytoplasmic granules, degranulated cells by the exocytosis of granules in the skin, immature MCs and the accumulation of histamine by MCs was evidenced by staining with 0.02% berberine sulphate, toluidine blue, and 2.5% Safranin-O (S-O) respectively. The mast cells were quantified in 10 fields per section, using a 40X objective on an ZEISS-AXIOSKOP2 microscope at the Immunomorphology Laboratory, IBILCE-UNESP, using an image analyzer (AXIOVISION software, Oberkkochen, Germany, version 4.8). Values are shown as mean ± standard error of the mean (SD) number of cells per mm^2^.

#### 2.5.2. Immunohistochemical Analysis

Representative sections from each sample were prepared for immunohistochemical staining. This involved deparaffinization with xylene and rehydration using a graded series of ethanol solutions. Antigen retrieval was performed by heating the sections at 96 °C for 30 min. Endogenous peroxide activity was blocked using 30% hydrogen peroxide for 15 min, followed by blocking with 5% BSA/PBS. The sections were then incubated with primary anti-rabbit NLRP3 clone SC06-23 antibodies 1:500 (#MA5-32255 ThermoFisher Scientific, Frederick, MD, USA). Negative controls were included by incubating some sections with 5% BSA instead of the primary antibody. After washing, the sections were incubated with a secondary biotinylated antibody (Histostatin^®^ Bulk Kit, Invitrogen, Frederick, MD, USA). Positive staining was detected using a peroxidase-conjugated streptavidin complex, and the color was developed using the DAB Substrate Kit (Invitrogen, Frederick, MD, USA). Hematoxylin was used for counterstaining the sections.

### 2.6. Multiplex Assay

To quantify the inflammatory mediator IL-1β, we used a LUMINEX xMAP MAGPIX multiplex instrument (Millipore Corporation, Billerica, MA, USA). The tissues were macerated in liquid nitrogen and placed in clean, 1.5 mL tubes, to which 500 µL of a solution containing protease inhibitor cocktail (GE Healthcare, Amersham, UK) and Tween 20 (1 µL) (Sigma-Aldrich, Poole, Dorset, UK) was added. The samples were incubated for 1 h at 4 °C under constant agitation and then centrifuged at 21,000× *g* for 10 min at 4 °C. The protein concentration in the supernatant was measured using a Bradford assay (Biorad, Hemel Hempsted, UK). Antibody beads, controls, wash buffer, serum matrix, and standards were prepared following the manufacturer’s instructions (MILLIPLEX HCYTOMAG-60K kit) (Merck, Millipore, Germany). A further 200 µL of wash buffer was added to each well of a magnetic 96-well plate and mixed on a shaker for 10 min. The wash buffer was decanted and 25 µL of standards, controls and samples were added to the wells. Next, 25 µL of assay buffer was added to the samples, and 25 µL of serum matrix was added to the standards. Finally, 25 µL of magnetic beads (coated with a specific capture antibody) was added to all wells and incubated overnight at 4 °C on a shaker. The next day, the plate was washed with wash buffer and incubated with 25 µL of detection antibodies for 1 h on a shaker. Next, 25 µL of streptavidinphycoerythrin was added to each well and incubated for 30 min on a shaker. The plate was washed and incubated with 150 µL of drive fluid for 5 min on a shaker. Finally, the plate was analyzed using MAGPIX with xPONENT software (Millipore Corporation, Billerica, MA, USA, version 3.5).

### 2.7. In Silico Analysis

A publicly available transcriptome dataset was selected from the transcriptome dataset of the Gene Expression Omnibus (GEO) repository, acessed on 28 May 2022: GSE182906 https://www.ncbi.nlm.nih.gov/geo/query/acc.cgi?acc=GSE182906. In this study, a total of 22 female db/db mice with type 2 diabetes and eight female C57BL/6J mice were included. Two full-thickness wound injuries were induced, and the mice were divided into the following groups: db/db mice (C57BLKS/J Iar-+Leprdb/+Leprdb), and wild type (WT) [27]. The individual datasets were analyzed using freely available algorithms from the GEO2R tool (available at http://www.ncbi.nlm.nih.gov/geo/geo2r/, accessed on 28 May 2023). This tool enabled the comparison of sample groups within the GEO series, allowing the identification of differentially expressed genes based on the experimental conditions. GEO2R was applied to detect Il-1β, Casp1 (caspase-1), Nlrp3, *Tpsab1* (tryptase alpha/beta 1), and *Tpsb2* (tryptase beta 2) genes under different experimental conditions.

### 2.8. Statistical Analysis

The obtained results underwent descriptive analysis and assessment of normality. Since the data followed a normal distribution, a two-way analysis of variance (ANOVA) was conducted using the two-way ANOVA test with Tukey’s post-test. All values were presented as mean ± standard deviation (SD), and *p*-values less than 0.05 were considered statistically significant. The analysis was performed using GraphPad Prism software, version 9.5.1. Sample size was determined based on a power analysis using preliminary data. Assuming an α (two-tailed) of 0.05 and a desired power (1–β) of 0.8, we calculated that a group size of 5 animals would be sufficient to detect at least 50% difference in key outcomes (such as mast cell counts or IL-1β levels between treated and untreated diabetic groups) with ~80% power. This effect size was estimated from pilot experiments and the literature reports of similar interventions.

## 3. Results

### 3.1. Histological Analysis

**Figure 1:**
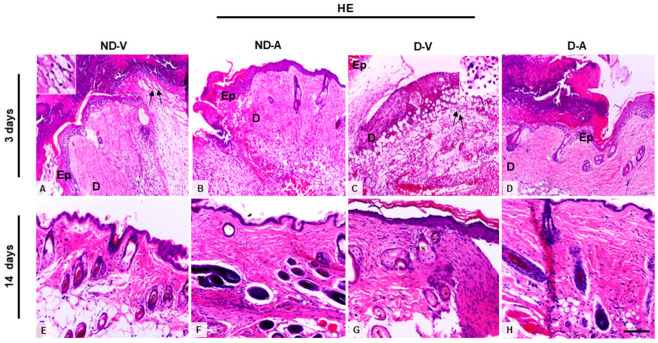
Histological analysis of the wound healing process (representative images of days 3 and 14). (**A**,**C**,**E**,**G**) Inflammatory influx in the ND-V and D-V groups. (**B**,**D**) Reduction of inflammatory infiltrate in the ND-A and D-A groups (day 3) and (**F**,**H**) improvement in tissue regeneration after 14 days of hydrogel treatment. Arrows indicate inflammatory influx in the ND-V and D-V groups. Inset shows a higher magnification of the indicated region, highlighting the inflammatory cells. Hematoxylin and eosin (HE) staining. Bars: 20 µm. Ep, epidermis; D, dermis. Non-diabetic vehicle hydrogel (ND-V); Non-diabetic Ac_2–26_ hydrogel (ND-A); Type 1 diabetes vehicle hydrogel (D-V) and Type 1 diabetes Ac_2–26_ hydrogel (D-A), (n = 5 animals/group).

Histological analysis of skin treated with the Ac_2–26_ hydrogel (Figure 1A) indicated a reduced influx of inflammatory cells on the third day of treatment. Additionally, we observed an improvement in the healing process after 14 days, indicating the efficacy of the hydrogel in promoting tissue regeneration and modulating the inflammatory response.

### 3.2. Ac_2–26_ Hydrogel Modulates Mast Cell Recruitment

**Figure 2:**
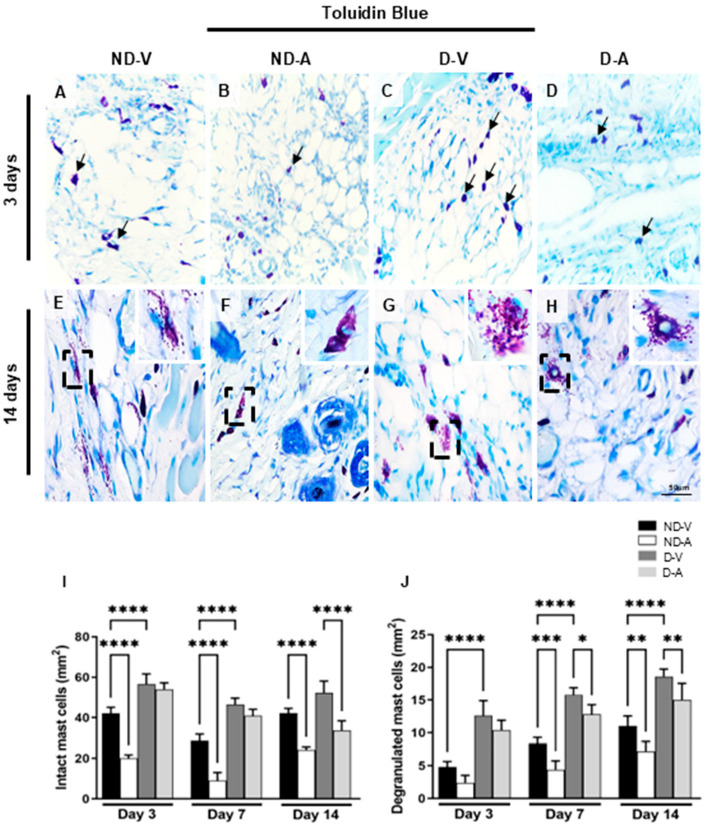
Quantification of mast cells in the healing process (representative images of days 3 and 14). Intact (arrows) (**A**–**D**) and degranulated mast cells (inset) (**E**–**H**). Quantification of intact (**I**), degranulated (**J**) mast cells. Staining: Toluidine blue. Bars: 50 μm. Non-diabetic vehicle hydrogel (ND-V); Non-diabetic Ac_2–26_ hydrogel (ND-A); Type 1 diabetes vehicle hydrogel (D-V) and Type 1 diabetes Ac_2–26_ hydrogel (D-A), (n = 5 animals/group). * *p* ≤ 0.05, ** *p* ≤ 0.01, *** *p* ≤ 0.001 and **** *p* ≤ 0.0001 versus treated animals and/or non-diabetic vehicle hydrogel.

Mast cells (MCs) were identified based on the metachromatic staining of cytoplasmic granules using toluidine blue (Figure 2A–H) and quantified at different stages of the healing process (Figure 2I,J). In non-diabetic animals, treatment with the Ac_2–26_ hydrogel (ND-A) significantly reduced the number of intact mast cells on days 3 and 14 compared with the non-diabetic vehicle group (ND-V). In diabetic animals, a reduction in intact mast cells was observed in the treated group (D-A) compared with the diabetic vehicle group (D-V), but only on day 14. Additionally, the D-V group showed significantly more mast cells than ND-V at all time points, suggesting increased inflammation under diabetic conditions.

Quantification of degranulated mast cells revealed higher numbers in ND-V compared to D-V at all time points. Treatment with Ac_2–26_ hydrogel reduced degranulation in both ND-A (vs. ND-V) and D-A (vs. D-V) on day 14, further indicating its anti-inflammatory effect.

### 3.3. Safranin-Positive Mast Cells Reduction After Ac_2–26_ Hydrogel Treatment

**Figure 3:**
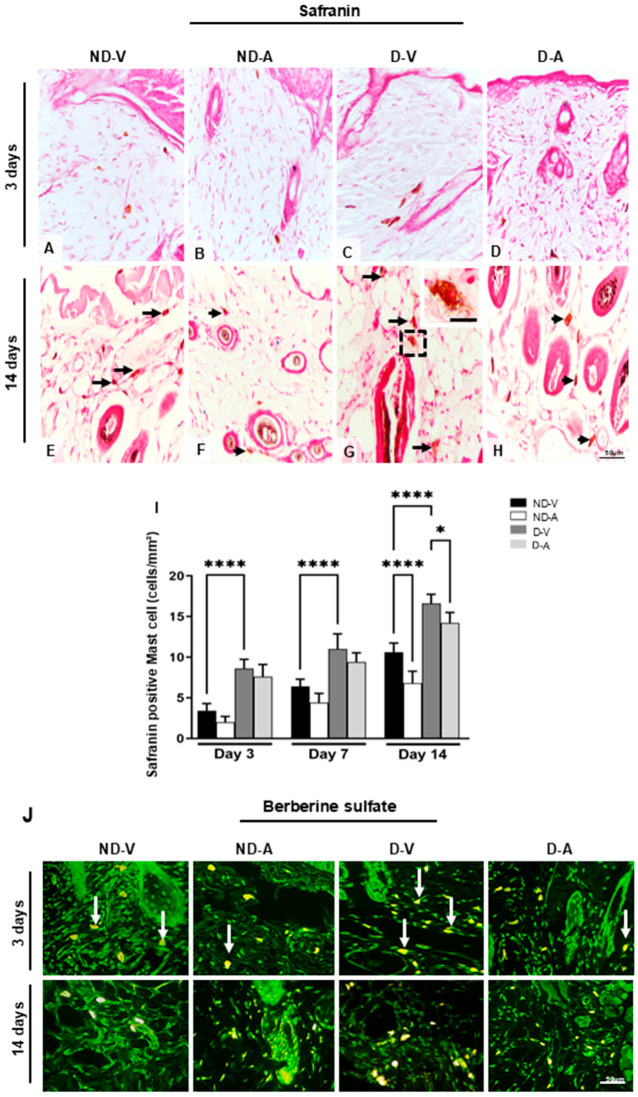
Quantification of mast cells during the healing process. Safranin staining (representative images from days 3 and 14) showing mast cells (**A**–**H**). Black arrows indicate mast cells (**E**–**H**), with an enlarged view of a mast cell in the inset (**G**). An increase in mast cell numbers is observed in (**E**,**G**), while a reduction after treatment is shown in (**F**,**H**). Quantification of safranin-positive mast cells is presented in (**I**). Berberine sulfate staining reveals mast cell granules with intense fluorescence in the ND-V and D-V groups (white arrows), and weaker fluorescence in the ND-A and D-A groups (**J**). Stainings: safranin and berberine sulfate. Scale bars: 20 μm (**A**–**I**) and 50 μm (**J**). Non-diabetic vehicle hydrogel (ND-V); Non-diabetic Ac_2–26_ hydrogel (ND-A); Type 1 diabetes vehicle hydrogel (D-V) and Type 1 diabetes Ac_2–26_ hydrogel (D-A), (n = 5 animals/group). * *p* ≤ 0.05 and **** *p* ≤ 0.0001 versus treated animals and/or non-diabetic vehicle hydrogel.

The mast cells’ metachromasia indicated the presence of highly sulfated glycosaminoglycans such as heparin. The quantification of safranin-positive mast cells showed an increase in the group ND-V in relation to group D-V on days 3 and 14 (Figure 3A–I) and a reduction in the numbers of these cells after treatment with Ac_2–26_ hydrogel in the ND-A and D-A groups after 14 days (Figure 3I).

Safranin staining indicated the presence of highly sulfated glycosaminoglycans (e.g., heparin) in mast cells. Quantification revealed higher numbers of safranin-positive MCs in ND-V compared with D-V on days 3 and 14 (Figure 3A–I). Treatment with Ac_2–26_ hydrogel resulted in a significant reduction in safranin-positive MCs in the ND-A and D-A groups on day 14.

The presence of heparin was further confirmed by berberine sulfate staining (Figure 3J), which showed intense staining in the ND-V and D-V groups, particularly on day 3. In contrast, mast cells in the ND-A and D-A groups were negative or weakly stained, suggesting that the Ac_2–26_ hydrogel may have reduced mast cell activation and granule content.

### 3.4. Decreased IL-1β Production After Ac_2–26_ Hydrogel Treatment in the Dermis

**Figure 4:**
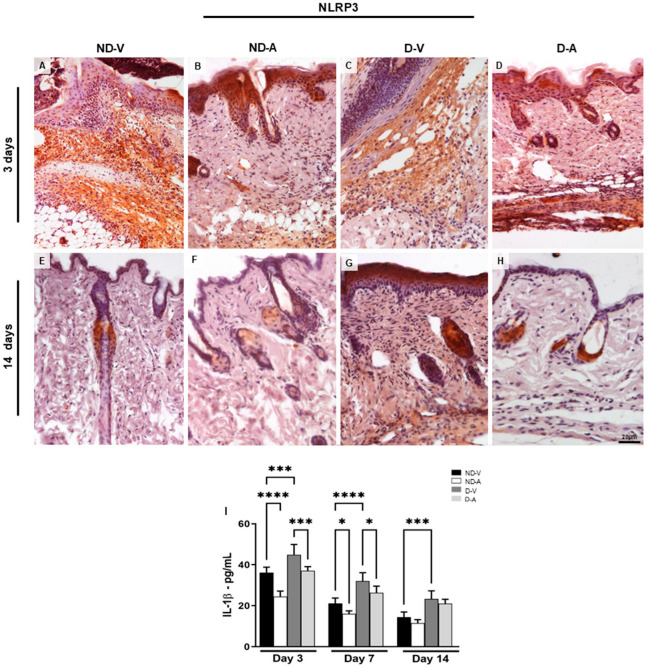
Expression of NLRP3 in the dermis and dosage of IL-1β mediator levels in the healing process. Immunohistochemistry (representative images of days 3 and 14) of NLRP3 positive cells (**A**–**H**). The inset shows the immunostained cell in detail. Bars: 20 µm. Levels of IL-1β (**I**). The data show means ± S.D of pg per mL of mediator (n = 5 animals/group), obtained using a multiplex assay for IL-1β quantification. Non-diabetic vehicle hydrogel (ND-V); Non-diabetic Ac_2–26_ hydrogel (ND-A); Type 1 diabetes vehicle hydrogel (D-V) and Type 1 diabetes Ac_2–26_ hydrogel (D-A). * *p* ≤ 0.05, *** *p* ≤ 0.001 and **** *p* ≤ 0.0001 versus treated animals and/or non-diabetic vehicle hydrogel.

Topical treatment with Ac_2–26_ hydrogel reduced the inflammatory response during skin healing, characterized by a reduction in the pro-inflammatory mediator interleukin 1β (IL-1β). The IL-1β dosage (Figure 4I) showed a reduction of this cytokine after 3 days of treatment with the Ac_2–26_ hydrogel in the ND-A and D-A groups. Furthermore, IL-1β increased in the ND-V and D-V groups in all evaluated periods.

Considering that the NLRP3 receptor is associated with IL-1β release during inflammatory processes [11,12], we investigated its expression under different experimental conditions. The immunoreactivity of NLRP3 was assessed by immunohistochemistry (Figure 4A–H). Skin samples from the ND-V and D-V groups exhibited strong NLRP3 immunoreactivity, particularly in regions with inflammatory cell infiltration (Figure 4A,C), whereas treatment with the Ac_2–26_ hydrogel resulted in weaker immunostaining (Figure 4B,D). After 14 days, reduced NLRP3 immunoreactivity persisted in the groups treated with the Ac_2–26_ hydrogel (Figure 4F,H).

Topical application of Ac_2–26_ hydrogel reduced inflammation during wound healing, as evidenced by lower levels of IL-1β (Figure 4I). Significant reductions were observed in ND-A and D-A groups on day 3, while IL-1β remained elevated in the ND-V and D-V groups across all time points.

### 3.5. In Silico Analyses

**Figure 5:**
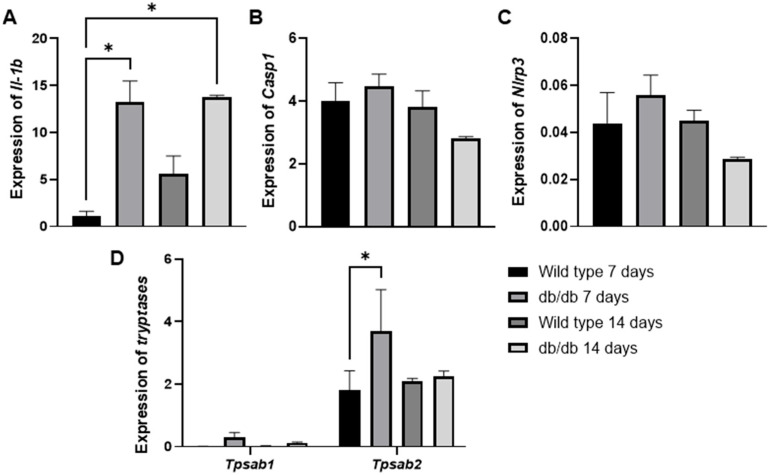
The mRNA expression levels of *Il1-b* (**A**), *Casp1* (**B**), *Nlrp3* (**C**), *Tpsab1*, and *Tpsb2* (**D**) in spontaneous diabetic db/db animal skin lesions were analyzed using in silico analysis based on the publicly available transcriptome data from the GSE182906 study. * *p* ≤ 0.05 versus healthy wild-type C57BL/6J animals. Statistical analysis was performed using one-way ANOVA.

To further support these findings, we evaluated the GSE182906 dataset, which included a microarray profile of skin lesions from spontaneous diabetic db/db animals (C57BLKS/J Iar-+Leprdb/+Leprdb) compared with healthy wild-type C57BL/6J animals (Figure 5A–D). In our analysis, we observed an increase in *Il1b* (* *p* < 0.01) gene expression in diabetic animals at 3 and 14 days compared with healthy wild-type animals at 3 days (Figure 5A). Additionally, we examined two mast cell-specific genes encoding mast cell proteases (*Tpsb2*, *Tpsab1*) (Figure 5D). We found that only the *Tpsb2* gene was overexpressed at 3 days (* *p* < 0.05) in diabetic animals compared with the control (Figure 5D). Although we did not observe significant increases in *Casp1* (Figure 5B) or *Nlrp3* (Figure 5C), the findings of *Il-1b* and *Tpsb2* are interesting clues that support our findings, suggesting potentially high mast cell activity in early wound healing in diabetic animals.

## 4. Discussion

In this study, we explored the therapeutic potential of the Ac_2–26_ peptide formulated as a topical hydrogel to promote cutaneous healing in a diabetic setting, focusing on its immunomodulatory effects, particularly on mast cell dynamics and IL-1β expression. Our findings, supported by macroscopic, histological, immunohistochemical, and bioinformatic analyses, suggest that Ac_2–26_ fosters a more controlled inflammatory milieu, reinforcing its role as an anti-inflammatory and pro-resolving agent.

Consistent with previous literature, diabetic wounds exhibited marked mast cell hyperactivation, evidenced by increased dermal mast cell density and enhanced degranulation, along with elevated histamine content [2,6]. Treatment with Ac_2–26_ hydrogel mitigated these alterations, significantly reducing both mast cell degranulation and histamine levels, indicating a direct inhibitory effect of the peptide on mast cell activation [3,28]. These data align with earlier findings that AnxA1 and its mimetics suppress mast cell degranulation and histamine release in various inflammatory settings [29,30,31].

The attenuation of IL-1β expression following Ac_2–26_ treatment further underscores the relevance of mast cells in the chronic inflammatory landscape of diabetic wounds. IL-1β is a pivotal cytokine that amplifies tissue injury and impedes regeneration by enhancing leukocyte infiltration and inflammatory cascades [6,32]. While mast cells are prominent producers of IL-1β, given that numerous cell types including keratinocytes, M1 macrophages, and neutrophils are well-established sources of IL-1β in cutaneous wounds [18,27], the observed attenuation of IL-1β expression following *Ac_2–26_* treatment probably reflects the peptide’s broad immunomodulatory activity within the wound microenvironment, rather than a solely mast cell-specific mechanism [14].

This notion is corroborated by our previous experimental data showing that AnxA1 mimetics modulate macrophage activation and foster a pro-resolving environment [25]. Indeed, we observed in previous experiments that AnxA1 mimetics can alter macrophage activation status in wounds, contributing to a more pro-resolving environment [18]. Thus, the improved healing seen with *Ac_2–26_* may result from a combination of direct effects on mast cells and indirect effects on other immune cells. It is important to note that AnxA1′s beneficial effects in wound healing are likely to be multi-faceted. In addition to mast cells, AnxA1 can influence other inflammatory cells such as macrophages and neutrophils [15,16,27,33,34].

This complexity is further illustrated by our bioinformatic analyses, demonstrating significant downregulation of Il1b and Tpsb2 following Ac_2–26_ application; these are key markers of IL-1β activity and mast cell protease expression. Nevertheless, one important constraint in the present study is the use of an external transcriptomic dataset to explore gene expression changes. Although the selected dataset is well characterized and relevant to the diabetic wound context [35], it does not originate from the same experimental cohort used for our functional and histological evaluations. Additionally, due to limited tissue availability, we were unable to directly assess gene or protein expression of inflammatory mediators in our own samples. As such, our interpretation of IL-1β and NLRP3 pathway modulation should be considered exploratory. Nonetheless, the convergence of transcriptomic and histological findings provides a compelling rationale for future studies incorporating integrated gene and protein expression analyses performed directly on wounds from the same experimental model.

Moreover, our in silico analysis of publicly available gene expression data was focused on a defined set of inflammatory mediators relevant to mast cell biology (e.g., Il1b, Tpsb2). While informative, this targeted approach did not encompass a comprehensive survey of all cell-type-specific markers and pathways potentially affected by Ac_2–26_ treatment. As such, we recognize that broader shifts in gene expression profiles, particularly those involving other immune or stromal populations, may not have been captured in the present analysis. Accordingly, we cannot rule out the possibility that Ac_2–26_ also exerts regulatory effects on additional inflammatory pathways or cell types, such as macrophages, keratinocytes, or fibroblasts. These aspects merit further exploration through expanded transcriptomic profiling and single-cell resolution analyses to more fully characterize the molecular networks modulated during diabetic wound repair.

Although we assessed NLRP3 expression as a supplementary inflammasome-related marker, our results do not establish a causal relationship between inflammasome activation and mast cell activity in diabetic wounds. While the integration of immunohistochemical and transcriptomic data provided coherent mechanistic insights on the impact of *Ac_2–26_* on key inflammatory pathways, direct protein-level quantification of mediators such as IL-1β, NLRP3, and caspase-1 was beyond the timeframe of this current work. Future work incorporating targeted protein quantification will be important to further validate these findings. Nevertheless, the significance of the current results undoubtedly helps to define a forward-looking research agenda. Comprehensive molecular profiling, protein validation, and functional studies using genetically modified models will be pivotal in fully elucidating the role of AnxA1 signaling and mast cell involvement in chronic wound inflammation and its resolution [14,36].

Our findings implicate mast cells in the diabetic wound healing deficit and its improvement by *Ac_2–26_*; nevertheless, we cannot conclude causality without direct intervention. Future studies employing mast cell-deficient animal models or mast cell depletion strategies (such as knockout mice or targeted pharmacological depletion) will be crucial to definitively establish the extent to which mast cells drive IL-1β production and impair healing in diabetes. Such experiments would also clarify the direct contribution of mast cell modulation to the therapeutic effects of AnxA1 mimetics.

## 5. Limitation and Future Directions

While our findings offer valuable insights into the pro-resolving actions of the AnxA1-derived peptide Ac_2–26_ in diabetic wound healing, a few limitations must be acknowledged to help direct future studies.

First, although the data suggest a contributory role for mast cells, the absence of mast cell-deficient models or targeted depletion strategies precludes definitive conclusions regarding their causative involvement. Future studies employing cell-specific ablation or lineage-tracing approaches (e.g., knockout mice, Cre-lox/Cre—Dre targeting of mast cell lineages) will be essential to clarify the precise role of mast cells in IL-1β production and in mediating the therapeutic effects of AnxA1 mimetics.

Second, our transcriptomic analysis, while informative, was limited to select inflammatory mediators. This analysis was focused on a limited subset of inflammatory mediators (e.g., IL1b, Tpsb2), primarily related to mast cell activity. However, this targeted approach precluded a comprehensive, unbiased analysis of cell-type-specific signatures. As such, we cannot exclude the possibility that Ac_2–26_ influences additional immune and non-immune cell populations—such as keratinocytes, macrophages, or fibroblasts—that were not captured in our dataset. Future studies utilizing single-cell RNA sequencing or spatial transcriptomics approaches may offer a more comprehensive view on the cellular and molecular mechanisms modulated by AnxA1 signalling.

Third, it should be noted that our study utilized group sizes of n = 5 per group for most endpoints. While our power analysis indicated this number was sufficient to detect the observed differences, the study can be considered a pilot-scale investigation. Accordingly, the findings will need to be confirmed in larger cohorts to strengthen their generalizability.

Finally, although direct protein-level analyses of key mediators (e.g., IL-1β, NLRP3, caspase-1) were beyond the timeframe of this study, future investigations incorporating targeted quantification strategies will be valuable to reinforce the molecular pathways proposed.

Taken together, these considerations do not detract from the core findings but rather outline a clear and promising roadmap for future research. Comprehensive molecular profiling, protein validation, and mechanistic studies employing genetically modified models will be instrumental in fully delineating the role of AnxA1 signalling and mast cell biology in the context of chronic wound inflammation and its resolution.

## 6. Conclusions

The present findings demonstrate that topical administration of the Ac_2–26_ peptide significantly modulated the inflammatory milieu of diabetic skin wounds, attenuating activation of mast cells and reducing IL-1β expression, which are two hallmark features of chronic wound pathology. These effects were accompanied by improved histoarchitectural indicators of repair, supporting the notion that AnxA1 mimetics may promote resolution of inflammation and tissue regeneration in diabetes-impaired healing.

While mast cells emerge as central players in this context, their role should be interpreted within the broader immunological framework of wound pathophysiology. Our results suggest a significant role for mast cells in sustaining inflammation and mediating the beneficial actions of Ac_2–26_; however, definitive causality cannot be ascribed. Indeed, the involvement of other cell types such as macrophages, neutrophils, and keratinocytes cannot be excluded and is likely to have contributed to the observed therapeutic effects.

Although the data are strongly suggestive, they do not yet establish a direct causal relationship implicating mast cells as the sole or principal mediators of impaired healing response. Future studies employing cell-specific ablation strategies will be essential to dissect the precise cellular targets of AnxA1 signalling and to define the mechanistic hierarchy among the inflammatory mediators involved.

Taken together, our data advance the mechanistic understanding of AnxA1-based interventions in cutaneous wound repair and highlight Ac_2–26_ as a promising candidate for the development of targeted therapies to resolve chronic inflammation and restore tissue homeostasis in diabetic wounds.

## Data Availability

The original contributions presented in this study are included in the article. Further inquiries can be directed to the corresponding author.

## Abbreviations

AnxA1	Annexin A1
IL-1 β	Interleukin 1 β
NLRP3	NOD-like receptor containing a pyrin-3 domain protein
DM1	Type 1 diabetes
MCs	Mast cells
AGEs	Advanced glycation end products
CASP1	Caspase 1
ASC	Apoptosis-associates speck-like protein containing CARD
CAPS	Cryopyrin-associated periodic syndrome
PLA2	Phospholipase A2
AnxA1_2–26_	N-terminal peptide Ac_2–26_
SDP	Sulfadiazine
CMC	Carboxymethyl cellulose
STZ	Streptozotocin
NDvH	Non-diabetic mice treated with vehicle hydrogel
NDAnxH	Non-diabetic mice treated with AnxA1_2–26_ hydrogel
T1DMvH	Diabetic mice treated with vehicle hydrogel
T1DMAnxH	Diabetic mice treated with AnxA1_2–26_ hydrogel
GEO	Gene Expression Omnibus
WT	Wild type
TPSAB1	Tryptase Alpha/Beta 1
TPSB2	Tryptase Beta 2
C57BLKS/J Iar+Leprdb/+Leprdb	Spontaneous diabetic db/db animals
AnxA1^-/-^	Annexin A1-deficient mice
LPS	Lipopolysaccharide
